# Supplementary material for: Transcriptomic Insights into Mechanisms of Early Seed Maturation in the Garden Pea (Pisum sativum L.)
Source: Cells. 2020 Mar 23;9(3):779. doi: 10.3390/cells9030779 (PMC7140803; doi:10.3390/cells9030779)
Supplement: Supplementary file 1 [file cells-09-00779-s001.zip › Supplementary materials/FileS4.zip!/FileS3/TFs - Results/Summary Sheets - Modules/Correlation Cluster/Report.pdf]

Correlation Cluster

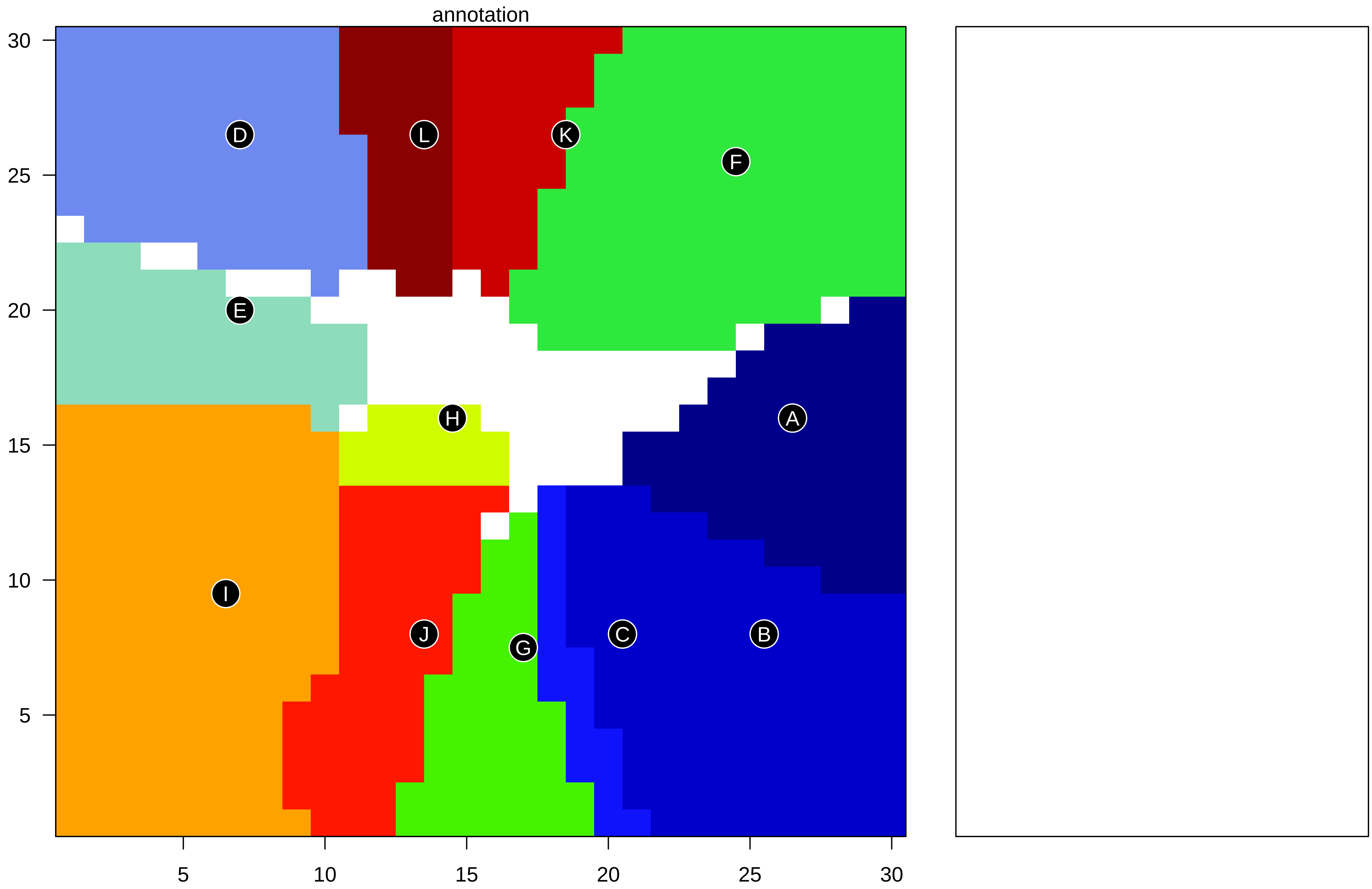

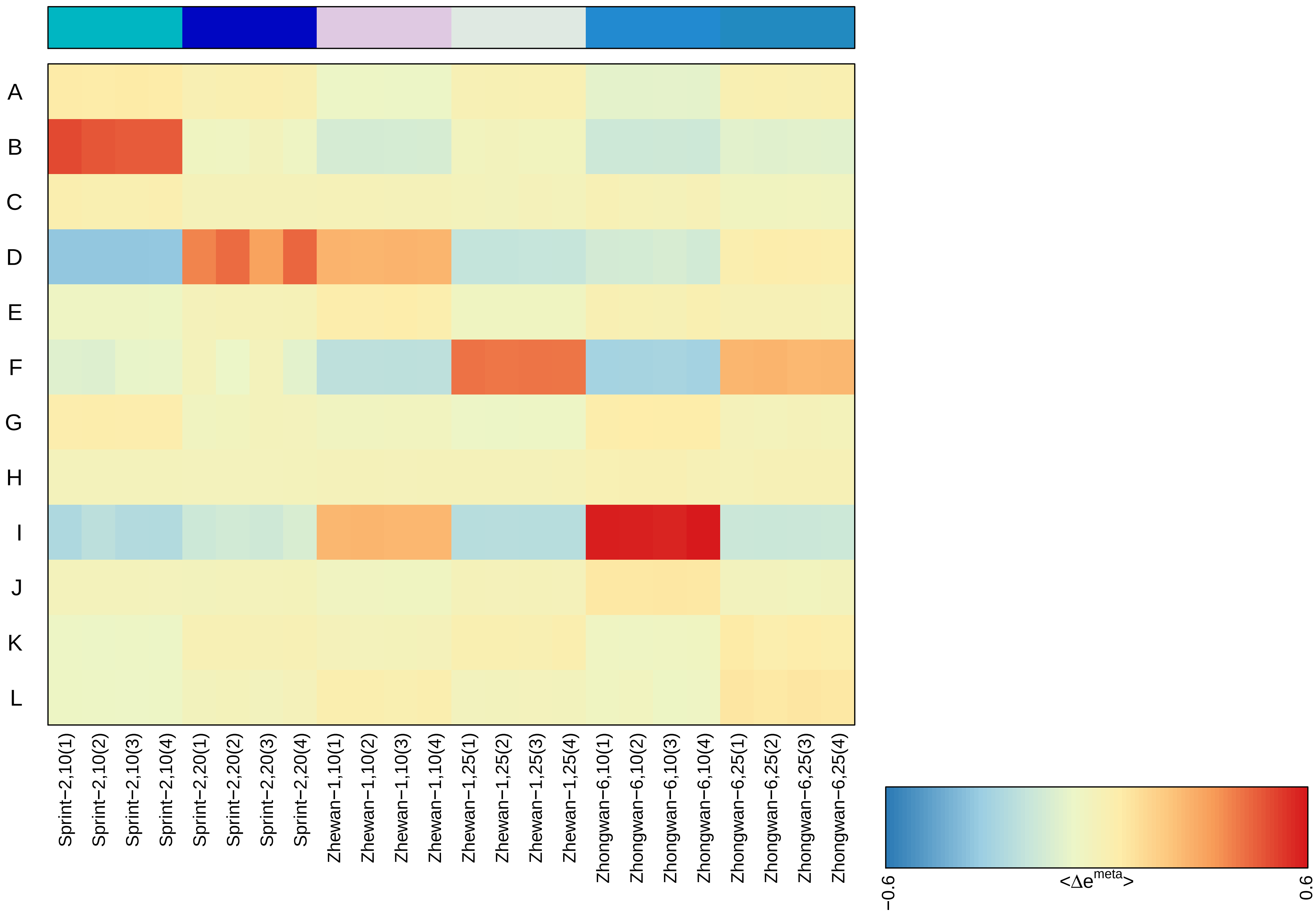
A
B
C
D
E
F
G
H
I
J
K
L
Sprint−2,10(1)
Sprint−2,10(2)
Sprint−2,10(3)
Sprint−2,10(4)
Sprint−2,20(1)
Sprint−2,20(2)
Sprint−2,20(3)
Sprint−2,20(4)
Zhewan−1,10(1)
Zhewan−1,10(2)
Zhewan−1,10(3)
Zhewan−1,10(4)
Zhewan−1,25(1)
Zhewan−1,25(2)
Zhewan−1,25(3)
Zhewan−1,25(4)
Zhongwan−6,10(1)
Zhongwan−6,10(2)
Zhongwan−6,10(3)
Zhongwan−6,10(4)
Zhongwan−6,25(1)
Zhongwan−6,25(2)
Zhongwan−6,25(3)
Zhongwan−6,25(4)
−0.6
$\langle \Delta e^{meta} \rangle$
0.6

Correlation Cluster

Spot Summary: A

metagenes = 72
genes = 78

<r> metagenes = 0.94
<r> genes = 0.47
beta: r2= 0.05 / log p= −0.56

samples with spot = 0 (0 %)

Overview Map

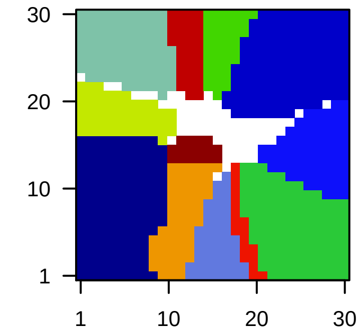

Spot

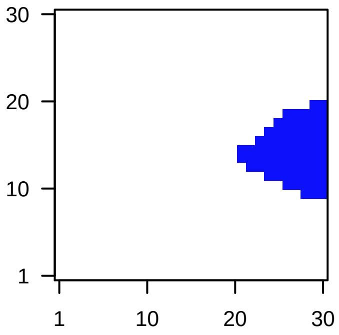

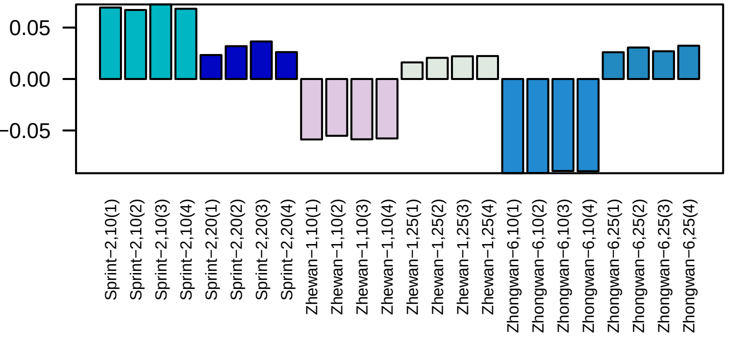

Spot Genelist

Rank	ID	max e	min e	r	Symbol	Description
1	TRINITY_DN1	0.54	−0.29	0.66		
2	TRINITY_DN9	0.34	−0.21	0.45		
3	TRINITY_DN1	0.32	−0.37	0.75		
4	TRINITY_DN3	0.32	−0.2	0.76		
5	TRINITY_DN2	0.28	−0.16	0.64		
6	TRINITY_DN3	0.26	−0.2	0.74		
7	TRINITY_DN3	0.23	−0.29	0.93		
8	TRINITY_DN3	0.22	−0.21	0.68		
9	TRINITY_DN1	0.22	−0.3	0.87		
10	TRINITY_DN7	0.21	−0.22	0.84		
11	TRINITY_DN1	0.21	−0.2	0.32		
12	TRINITY_DN1	0.21	−0.14	0.71		
13	TRINITY_DN8	0.21	−0.37	0.83		
14	TRINITY_DN1	0.19	−0.19	0.46		
15	TRINITY_DN2	0.19	−0.31	0.9		
16	comp39208_c	0.19	−0.03	0.38		
17	TRINITY_DN2	0.18	−0.09	0.51		
18	TRINITY_DN4	0.18	−0.15	0.92		
19	TRINITY_DN5	0.18	−0.09	0.77		
20	TRINITY_DN7	0.17	−0.1	0.78		

Correlation Cluster

Spot Summary: B

metagenes = 120
genes = 415

<r> metagenes = 0.96
<r> genes = 0.6
beta: r2= 0.53 / log p= −4.29

samples with spot = 4 (16.7 %)
Sprint−2(10) : 4 (100 %)

Overview Map

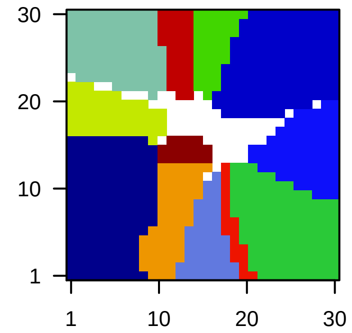

Spot

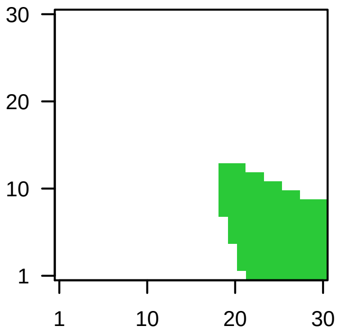

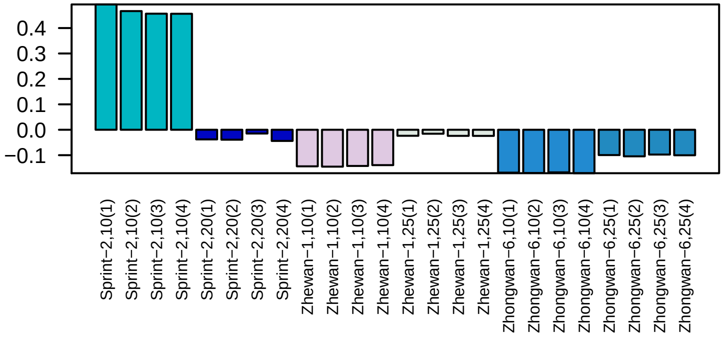

Spot Genelist

Rank	ID	max e	min e	r	Symbol	Description
1	TRINITY_DN5	8.61	−1.68	0.96		
2	TRINITY_DN2	7.6	−8.44	0.83		
3	TRINITY_DN1	7.51	−1.97	0.98		
4	TRINITY_DN1	4.85	−1.19	0.96		
5	TRINITY_DN1	4.67	−2.15	0.89		
6	TRINITY_DN6	4.43	−1.66	0.86		
7	TRINITY_DN9	3.91	−0.84	0.96		
8	TRINITY_DN7	3.84	−2.46	0.46		
9	TRINITY_DN1	3.43	−1.66	0.85		
10	TRINITY_DN1	3.36	−2.28	0.87		
11	TRINITY_DN6	3.29	−1.71	0.91		
12	TRINITY_DN1	3.27	−0.38	0.8		
13	TRINITY_DN7	3	−0.92	0.91		
14	TRINITY_DN2	2.98	−3.37	0.69		
15	TRINITY_DN1	2.98	−1.06	0.97		
16	TRINITY_DN7	2.89	−0.48	0.96		
17	TRINITY_DN3	2.65	−0.56	0.97		
18	TRINITY_DN1	2.6	−0.83	0.98		
19	TRINITY_DN5	2.52	−1.4	0.8		
20	TRINITY_DN5	2.4	−1.02	0.93		

Correlation Cluster

Spot Summary: C

metagenes = 18
genes = 55

<r> metagenes = 0.98
<r> genes = 0.55
beta: r2= 0.01 / log p= −0.17

samples with spot = 0 (0 %)

Overview Map

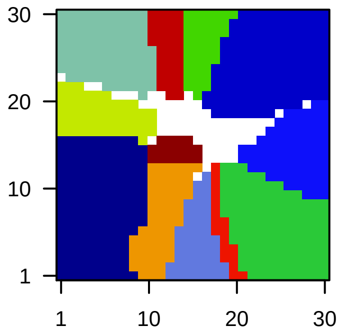

Spot

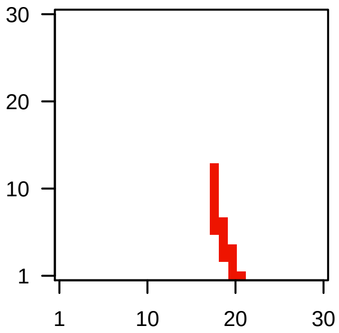

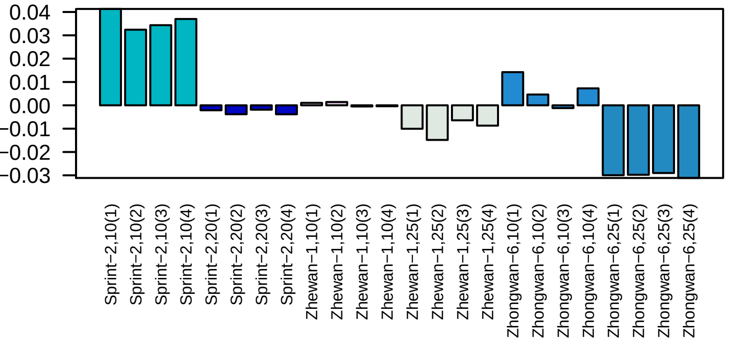

Spot Genelist

Rank	ID	max e	min e	r	Symbol	Description
1	TRINITY_DN5	0.27	−0.19	0.74		
2	TRINITY_DN4	0.19	−0.11	0.88		
3	TRINITY_DN5	0.19	−0.18	0.86		
4	TRINITY_DN5	0.16	−0.13	0.54		
5	TRINITY_DN1	0.16	−0.14	0.49		
6	TRINITY_DN2	0.15	−0.1	0.82		
7	TRINITY_DN2	0.14	−0.22	0.75		
8	TRINITY_DN8	0.13	−0.05	0.83		
9	TRINITY_DN1	0.12	−0.09	0.82		
10	TRINITY_DN1	0.12	−0.09	0.49		
11	TRINITY_DN6	0.11	−0.26	0.75		
12	TRINITY_DN9	0.11	−0.05	0.84		
13	TRINITY_DN3	0.1	−0.08	0.64		
14	TRINITY_DN9	0.09	−0.03	0.84		
15	TRINITY_DN6	0.09	−0.02	0.82		
16	TRINITY_DN5	0.08	−0.14	0.43		
17	TRINITY_DN9	0.08	−0.05	0.78		
18	TRINITY_DN1	0.07	−0.01	0.06		
19	TRINITY_DN2	0.06	−0.06	0.72		
20	TRINITY_DN3	0.06	−0.01	0.81		

Correlation Cluster

Spot Summary: D

metagenes = 90
genes = 220

<r> metagenes = 0.95
<r> genes = 0.47
beta: r2= 0.53 / log p= −4.31

samples with spot = 8 (33.3 %)
Sprint−2(20) : 4 (100 %)
Zhewan−1(10) : 4 (100 %)

Spot Genelist

Rank	ID	max e	min e	r	Symbol	Description
1	TRINITY_DN4	8.82	−8.66	0.91		
2	TRINITY_DN4	8.39	−5.76	0.93		
3	TRINITY_DN4	5.37	−4.32	0.97		
4	TRINITY_DN1	3.77	−2.56	0.82		
5	TRINITY_DN3	3.65	−1.82	0.74		
6	TRINITY_DN5	3.42	−2.06	0.87		
7	TRINITY_DN4	2.8	−1.11	0.78		
8	TRINITY_DN2	2.61	−1.75	0.84		
9	TRINITY_DN5	2.36	−2.06	0.8		
10	TRINITY_DN5	2.24	−1.8	0.86		
11	TRINITY_DN4	2.18	−1.82	0.79		
12	TRINITY_DN2	2.08	−1.08	0.91		
13	TRINITY_DN1	2.07	−0.94	0.74		
14	TRINITY_DN4	1.85	−1.11	0.73		
15	TRINITY_DN5	1.76	−0.46	0.71		
16	TRINITY_DN1	1.73	−1.06	0.86		
17	TRINITY_DN1	1.71	−1.55	0.7		
18	TRINITY_DN5	1.6	−1.18	0.59		
19	TRINITY_DN9	1.54	−0.51	0.45		
20	TRINITY_DN1	1.54	−0.9	0.98		

Overview Map

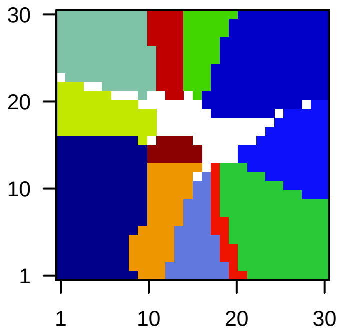

Spot

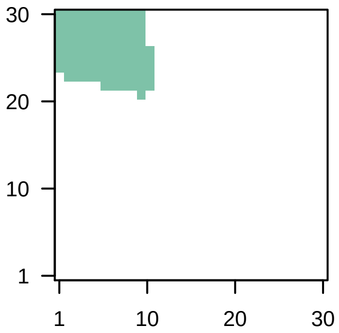

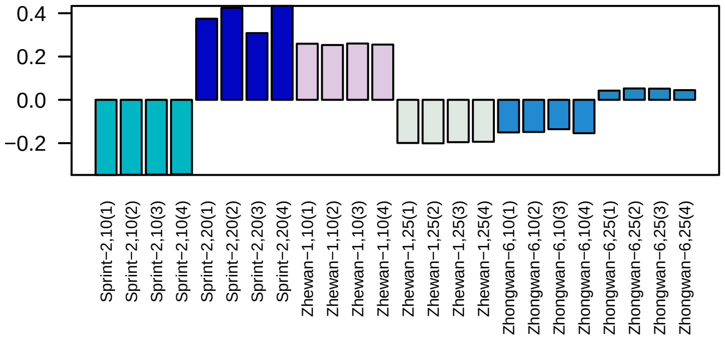

Correlation Cluster

Spot Summary: E

metagenes = 52
genes = 52

<r> metagenes = 0.95
<r> genes = 0.43
beta: r2= 0.03 / log p= −0.36

samples with spot = 0 (0 %)

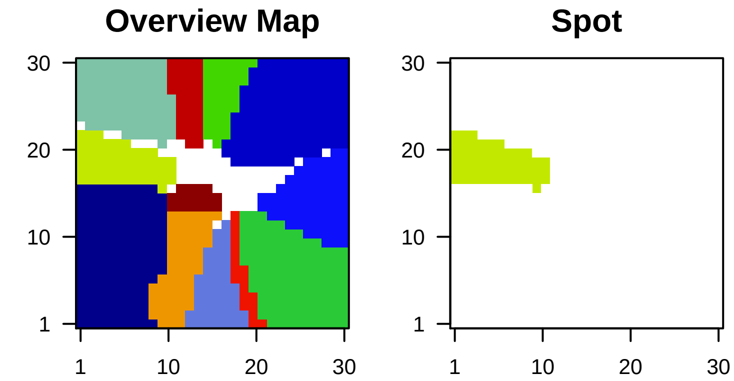

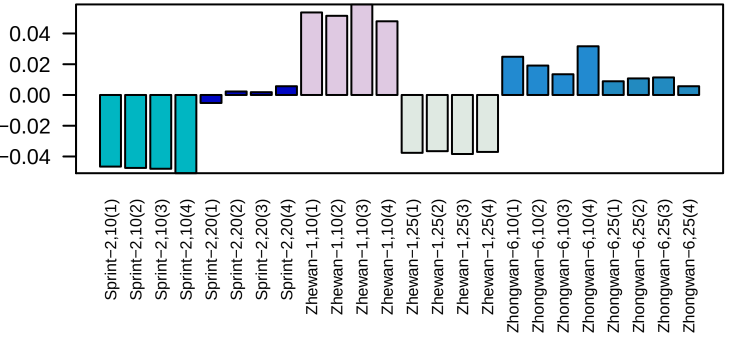

Spot Genelist

Rank	ID	max e	min e	r	Symbol	Description
1	TRINITY_DN7	0.29	−0.13	0.4		
2	TRINITY_DN4	0.26	−0.06	0.55		
3	TRINITY_DN1	0.26	−0.17	0.63		
4	TRINITY_DN3	0.21	−0.11	0.48		
5	TRINITY_DN5	0.2	−0.12	0.56		
6	TRINITY_DN8	0.19	−0.15	0.48		
7	comp11606_c	0.18	−0.05	0.67		
8	comp59039_c	0.18	−0.03	0.65		
9	TRINITY_DN6	0.17	−0.18	0.56		
10	TRINITY_DN5	0.17	−0.14	0.49		
11	TRINITY_DN2	0.16	−0.11	0.54		
12	TRINITY_DN3	0.16	−0.12	0.71		
13	TRINITY_DN3	0.14	−0.04	0.77		
14	TRINITY_DN1	0.14	−0.07	0.91		
15	TRINITY_DN2	0.14	−0.04	0.76		
16	TRINITY_DN5	0.13	−0.07	0.57		
17	TRINITY_DN1	0.13	−0.07	0.87		
18	TRINITY_DN4	0.13	−0.04	0.68		
19	TRINITY_DN2	0.13	−0.08	0.39		
20	TRINITY_DN5	0.12	−0.14	0.3		

Correlation Cluster

Spot Summary: F

metagenes = 139
genes = 338

<r> metagenes = 0.94
<r> genes = 0.5
beta: r2= 0.61 / log p= −5.18

samples with spot = 8 (33.3 %)
Zhewan−1(25) : 4 (100 %)
Zhongwan−6(25) : 4 (100 %)

Spot Genelist

Rank	ID	max e	min e	r	Symbol	Description
1	TRINITY_DN1	10.4	−3.02	0.84		
2	TRINITY_DN4	7.91	−7.93	0.93		
3	TRINITY_DN4	7.74	−4.91	0.78		
4	TRINITY_DN1	7.72	−3.91	0.76		
5	TRINITY_DN2	5.9	−1.92	0.75		
6	TRINITY_DN2	4.81	−3.98	0.96		
7	TRINITY_DN5	4.22	−2.39	0.98		
8	TRINITY_DN2	4.11	−3.63	0.88		
9	TRINITY_DN3	3.31	−3.6	0.56		
10	TRINITY_DN1	3.23	−1.54	0.91		
11	TRINITY_DN8	2.99	−2.22	0.84		
12	TRINITY_DN7	2.62	−3.66	0.57		
13	TRINITY_DN6	2.53	−0.84	0.88		
14	TRINITY_DN1	2.27	−1	0.95		
15	TRINITY_DN2	2.25	−1.48	0.56		
16	TRINITY_DN4	2.14	−0.47	0.56		
17	TRINITY_DN1	2.09	−1.85	0.77		
18	TRINITY_DN9	2.04	−0.73	0.82		
19	TRINITY_DN9	1.98	−1.08	0.89		
20	TRINITY_DN4	1.92	−4.32	0.65		

Overview Map

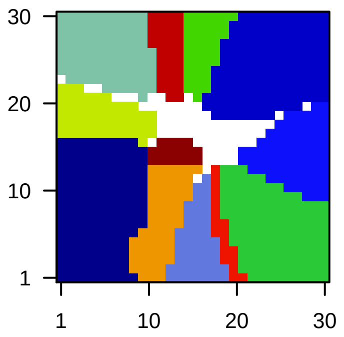

Spot

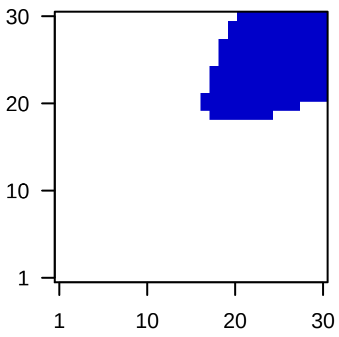

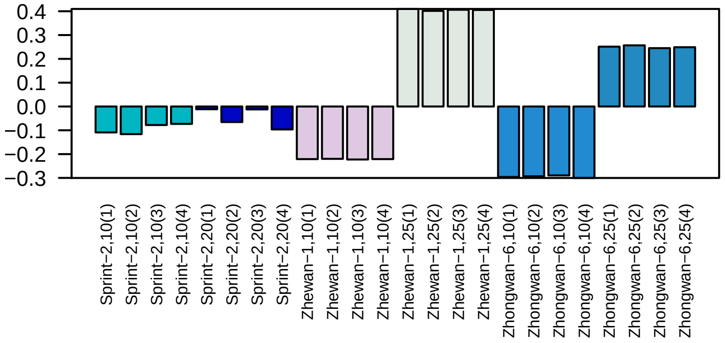

Correlation Cluster

Spot Summary: G

metagenes = 47
genes = 64

<r> metagenes = 0.93
<r> genes = 0.44
beta: r2= 0.03 / log p= −0.34

samples with spot = 0 (0 %)

Overview Map

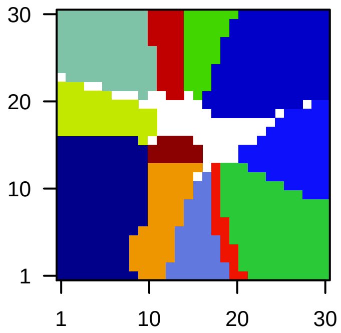

Spot

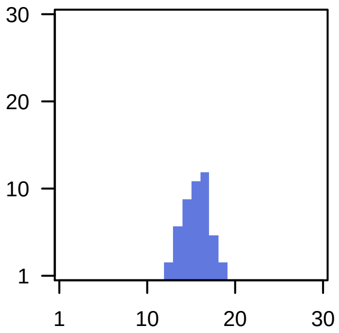

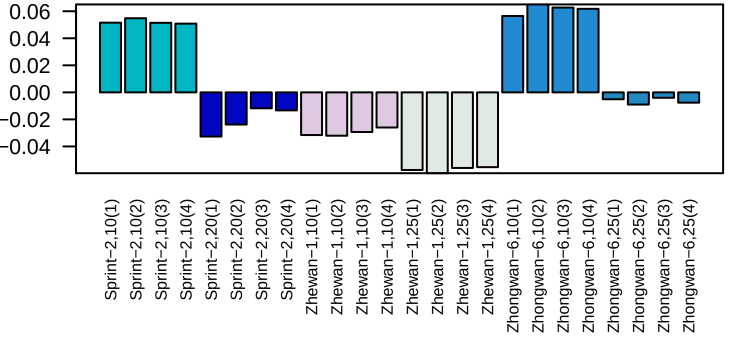

Spot Genelist

Rank	ID	max e	min e	r	Symbol	Description
1	TRINITY_DN7	0.92	−1.05	0.62		
2	TRINITY_DN5	0.45	−0.46	0.61		
3	TRINITY_DN1	0.25	−0.11	0.83		
4	TRINITY_DN3	0.25	−0.21	0.65		
5	TRINITY_DN3	0.23	−0.13	0.91		
6	TRINITY_DN9	0.22	−0.14	0.71		
7	TRINITY_DN4	0.21	−0.26	0.63		
8	TRINITY_DN1	0.21	−0.18	0.68		
9	TRINITY_DN5	0.21	−0.2	0.68		
10	TRINITY_DN5	0.2	−0.19	0.81		
11	TRINITY_DN1	0.2	−0.09	0.77		
12	TRINITY_DN5	0.19	−0.23	0.75		
13	TRINITY_DN4	0.18	−0.12	0.62		
14	TRINITY_DN8	0.18	−0.18	0.73		
15	TRINITY_DN1	0.17	−0.09	0.38		
16	TRINITY_DN3	0.17	−0.09	0.78		
17	TRINITY_DN5	0.15	−0.12	0.83		
18	TRINITY_DN1	0.15	−0.1	0.81		
19	TRINITY_DN1	0.14	−0.07	0.85		
20	TRINITY_DN1	0.14	−0.07	0.93		

Correlation Cluster

Spot Summary: H

metagenes = 16
genes = 45

<r> metagenes = 0.94
<r> genes = 0.71
beta: r2= 0 / log p= −0.08

samples with spot = 0 (0 %)

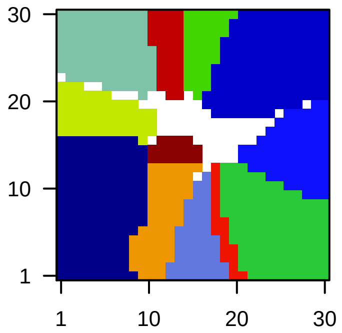

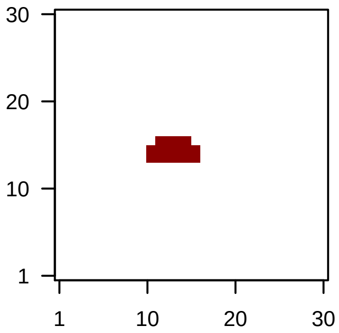

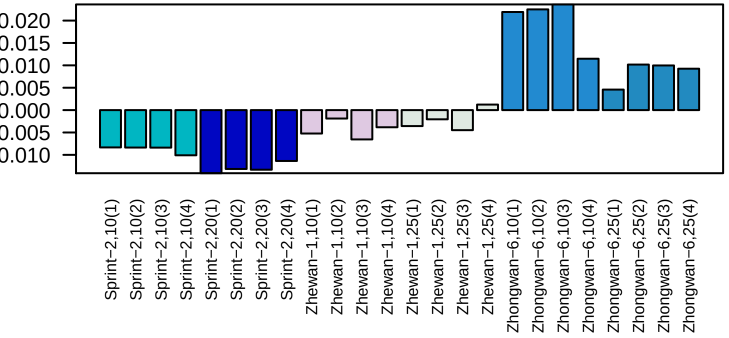

Spot Genelist

Rank	ID	max e	min e	r	Symbol	Description
1	TRINITY_DN9	0.23	−0.16	0.69		
2	TRINITY_DN3	0.16	−0.23	0.55		
3	TRINITY_DN4	0.13	−0.06	0.76		
4	TRINITY_DN3	0.07	−0.02	0.72		
5	TRINITY_DN3	0.05	−0.01	0.64		
6	TRINITY_DN9	0.05	−0.01	0.63		
7	TRINITY_DN8	0.05	−0.02	0.77		
8	TRINITY_DN3	0.04	−0.02	0.95		
9	TRINITY_DN5	0.04	−0.02	0.9		
10	TRINITY_DN7	0.04	−0.01	0.54		
11	TRINITY_DN1	0.04	−0.02	0.87		
12	TRINITY_DN2	0.03	−0.02	0.79		
13	TRINITY_DN1	0.03	−0.01	0.78		
14	TRINITY_DN1	0.03	−0.02	0.95		
15	TRINITY_DN2	0.03	−0.01	0.89		
16	TRINITY_DN1	0.03	−0.01	0.86		
17	TRINITY_DN2	0.03	−0.01	0.88		
18	TRINITY_DN5	0.03	−0.01	0.87		
19	TRINITY_DN2	0.03	−0.01	0.29		
20	TRINITY_DN9	0.03	−0.02	0.62		

Correlation Cluster

Spot Summary: I

metagenes = 149
genes = 336

<r> metagenes = 0.94
<r> genes = 0.58
beta: r2= 0.99 / log p= −Inf

samples with spot = 8 (33.3 %)
Zhewan−1(10) : 4 (100 %)
Zhongwan−6(10) : 4 (100 %)

Overview Map

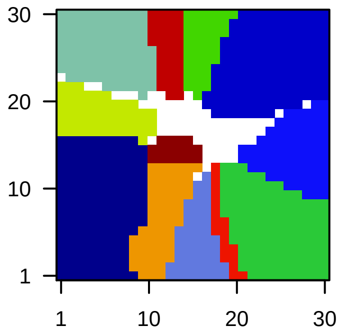

Spot

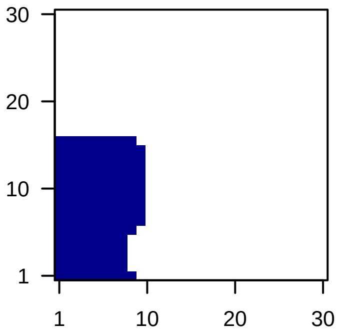

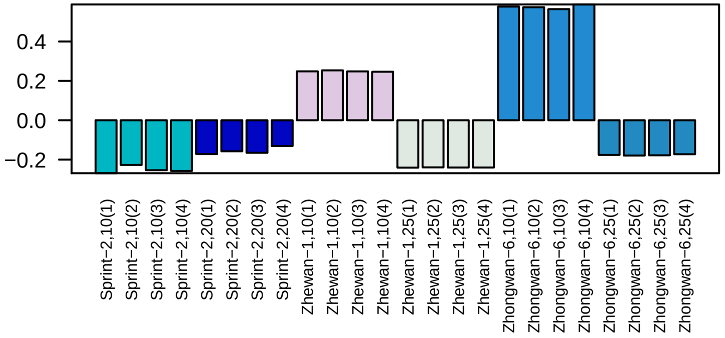

Spot Genelist

Rank	ID	max e	min e	r	Symbol	Description
1	TRINITY_DN1	13.12	−4.78	0.7		
2	TRINITY_DN7	11.07	−3.03	0.97		
3	TRINITY_DN1	8.39	−3.05	0.76		
4	TRINITY_DN1	6.17	−3.24	0.92		
5	TRINITY_DN4	5.77	−4.28	0.75		
6	TRINITY_DN3	5.76	−2.29	0.9		
7	TRINITY_DN1	5.25	−2.61	0.94		
8	TRINITY_DN1	4.57	−1.48	0.98		
9	TRINITY_DN2	4.53	−1.15	0.88		
10	TRINITY_DN3	4.41	−2.6	0.95		
11	TRINITY_DN2	4.05	−1.09	0.83		
12	TRINITY_DN3	3.87	−6.3	0.7		
13	TRINITY_DN3	3.49	−1.03	0.95		
14	TRINITY_DN7	3.48	−1.29	0.99		
15	TRINITY_DN2	3.3	−0.56	0.83		
16	TRINITY_DN2	3.29	−0.85	0.48		
17	TRINITY_DN1	3.26	−3.12	0.7		
18	TRINITY_DN1	2.87	−1.41	0.54		
19	TRINITY_DN2	2.8	−1.04	0.96		
20	TRINITY_DN8	2.78	−0.81	0.92		

Correlation Cluster

Spot Summary: J

metagenes = 59
genes = 163

<r> metagenes = 0.95
<r> genes = 0.64
beta: r2= 0.03 / log p= −0.36

samples with spot = 0 (0 %)

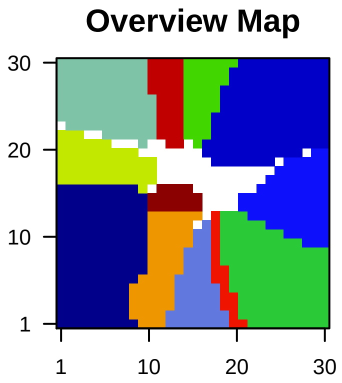

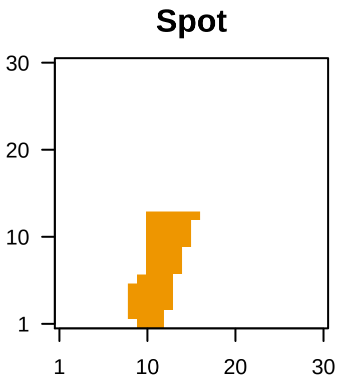

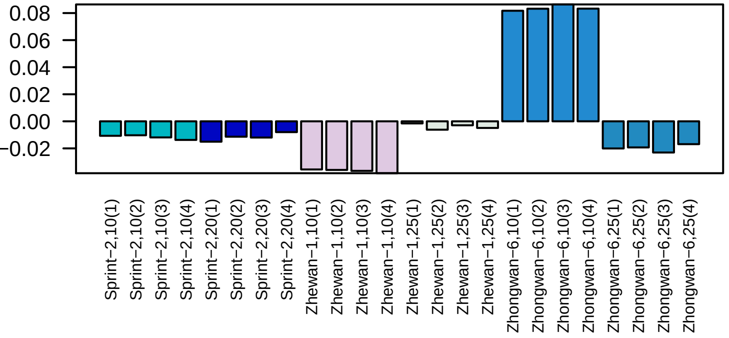

Spot Genelist

Rank	ID	max e	min e	r	Symbol	Description
1	TRINITY_DN9	1.02	−1.15	0.49		
2	TRINITY_DN3	0.48	−0.3	0.8		
3	TRINITY_DN1	0.4	−0.22	0.65		
4	TRINITY_DN1	0.39	−0.18	0.53		
5	TRINITY_DN3	0.35	−0.05	0.74		
6	TRINITY_DN5	0.33	−0.12	0.94		
7	TRINITY_DN2	0.32	−0.09	0.88		
8	TRINITY_DN1	0.32	−0.22	0.69		
9	TRINITY_DN2	0.32	−0.07	0.85		
10	TRINITY_DN4	0.3	−0.1	0.72		
11	TRINITY_DN4	0.29	−0.14	0.85		
12	comp48313_c	0.29	−0.03	0.64		
13	comp48132_c	0.28	−0.04	0.91		
14	TRINITY_DN3	0.28	−0.06	0.92		
15	comp32197_c	0.27	−0.19	0.64		
16	TRINITY_DN4	0.27	−0.03	0.69		
17	TRINITY_DN7	0.27	−0.07	0.85		
18	TRINITY_DN9	0.27	−0.2	0.77		
19	TRINITY_DN1	0.26	−0.11	0.66		
20	TRINITY_DN1	0.26	−0.04	0.81		

Correlation Cluster

Spot Summary: K

metagenes = 38
genes = 47

<r> metagenes = 0.97
<r> genes = 0.42
beta: r2= 0.03 / log p= −0.39

samples with spot = 0 (0 %)

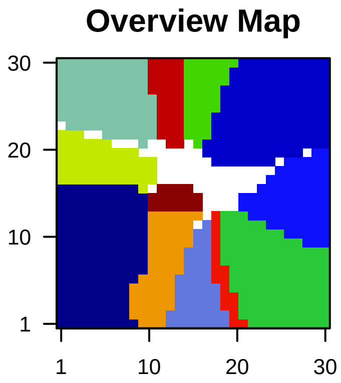

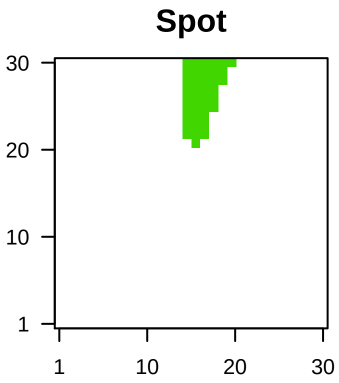

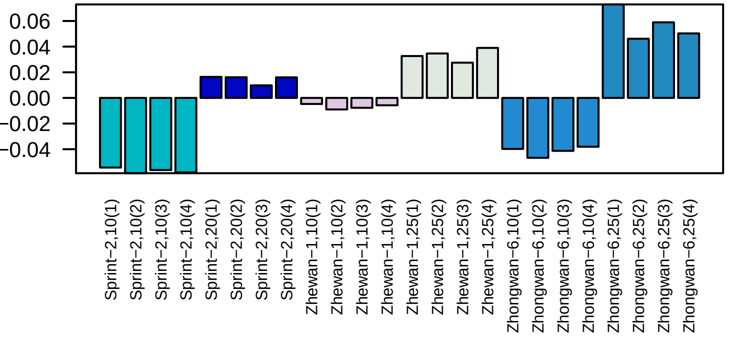

Spot Genelist

Rank	ID	max e	min e	r	Symbol	Description
1	TRINITY_DN9	0.37	−0.28	0.42		
2	TRINITY_DN2	0.28	−0.05	0.55		
3	TRINITY_DN7	0.25	−0.21	0.73		
4	TRINITY_DN5	0.23	−0.1	0.26		
5	TRINITY_DN5	0.23	−0.22	0.42		
6	TRINITY_DN1	0.23	−0.12	0.66		
7	TRINITY_DN3	0.22	−0.17	0.64		
8	TRINITY_DN2	0.21	−0.15	0.1		
9	TRINITY_DN3	0.2	−0.09	0.83		
10	TRINITY_DN1	0.19	−0.12	0.64		
11	comp40944_c	0.18	−0.02	0.49		
12	TRINITY_DN1	0.18	−0.19	0.7		
13	TRINITY_DN9	0.17	−0.06	0.46		
14	TRINITY_DN2	0.17	−0.14	0.75		
15	TRINITY_DN5	0.17	−0.15	0.62		
16	TRINITY_DN1	0.16	−0.1	0.86		
17	TRINITY_DN5	0.15	−0.13	0.6		
18	TRINITY_DN2	0.15	−0.05	0.72		
19	TRINITY_DN6	0.15	−0.05	0.75		
20	TRINITY_DN6	0.14	−0.19	0.75		

Correlation Cluster

Spot Summary: L

metagenes = 33
genes = 55

<r> metagenes = 0.97
<r> genes = 0.44
beta: r2= 0.02 / log p= −0.33

samples with spot = 0 (0 %)

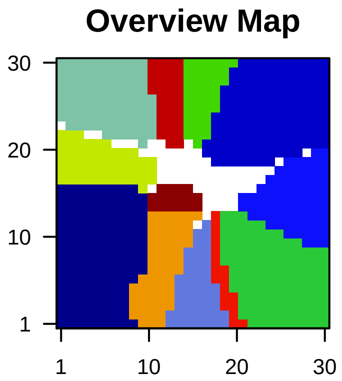

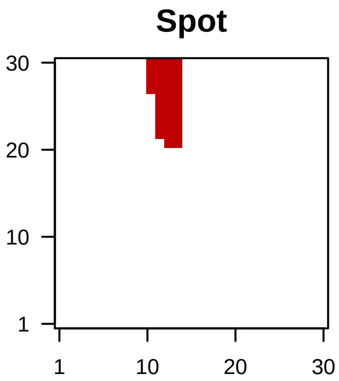

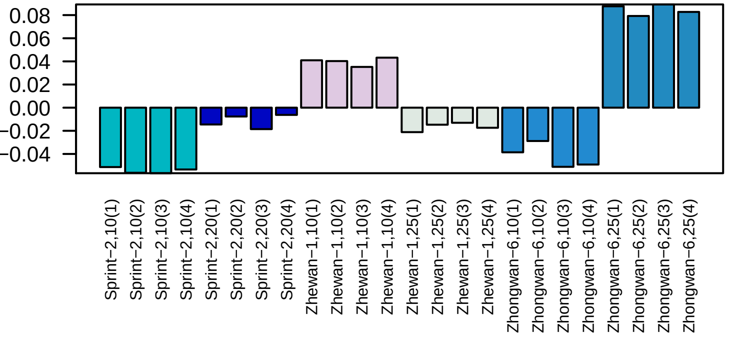

Spot Genelist

Rank	ID	max e	min e	r	Symbol	Description
1	TRINITY_DN5	0.41	−0.46	0.68		
2	TRINITY_DN1	0.36	−0.3	0.58		
3	TRINITY_DN3	0.31	−0.27	0.84		
4	TRINITY_DN6	0.31	−0.25	0.44		
5	TRINITY_DN2	0.29	−0.13	0.87		
6	TRINITY_DN7	0.29	−0.31	0.68		
7	TRINITY_DN1	0.28	−0.2	0.82		
8	TRINITY_DN5	0.28	−0.21	0.42		
9	comp24437_c	0.27	−0.1	0.9		
10	TRINITY_DN1	0.26	−0.38	0.38		
11	TRINITY_DN2	0.25	−0.08	0.91		
12	TRINITY_DN4	0.23	−0.11	0.89		
13	TRINITY_DN4	0.21	−0.15	0.73		
14	TRINITY_DN3	0.19	−0.18	0.56		
15	TRINITY_DN6	0.18	−0.19	0.47		
16	TRINITY_DN1	0.16	−0.07	0.87		
17	TRINITY_DN3	0.16	−0.02	−0.09		
18	TRINITY_DN8	0.16	−0.12	0.66		
19	TRINITY_DN2	0.15	−0.13	0.48		
20	TRINITY_DN2	0.15	−0.05	0.8		
